# Supplementary material for: Does Vaccinating against Influenza in a Given Epidemic Season Have an Impact on Vaccination in the Next Season: A Follow-Up Study
Source: Int J Environ Res Public Health. 2022 Jun 29;19(13):7976. doi: 10.3390/ijerph19137976 (PMC9265947; doi:10.3390/ijerph19137976)
Supplement: Supplementary file 1 [file ijerph-19-07976-s001.zip › ijerph-1769395-supplementary.pdf]

Does vaccinating against influenza in a given epidemic season have an impact on vaccination in the next season: a follow-up study

CODE: ☐☐☐

Date ……………………………………..

I. DEMOGRAPHIC DATA AND MEDICAL HISTORY

1. **Age:** ☐☐ [years]
2. **Gender:**

☐ Female ☐ Male

3. **Marital status:**

1. ☐ married
2. ☐ widow(er)
3. ☐ maiden/bachelor
4. ☐ divorced
5. ☐ free relationship
6. ☐ other (please, specify)................

4. **Source of income:**

1. ☐ employed
2. ☐ pensioner
3. ☐ disability sickness allowance
4. ☐ unemployed
6. ☐ other (please, specify)?............................

5. **Have you got a chronic disease?**

1. ☐ yes
2. ☐ no
3. ☐ I do not want to reveal

6. If „yes” (question 5) - please, specify:

1. ☐ diabetes
2. ☐ chronic kidney disease
3. ☐ chronic heart disease
4. ☐ chronic liver disease
5. ☐ cancer
6. ☐ other (please, specify)………………………………

II. DATA ON INFLUENZA VACCINATION

7. Did you get the flu shot last season?

1. ☐ yes
2. ☐ no
3. ☐ I don’t remember

8. Have you been vaccinated in previous seasons (more than a year ago)?

1. ☐ yes - how many times?...........
2. ☐ no
3. ☐ I don’t remember

9. Why did you decide to get vaccinated this season? (you can choose several options)

1. ☐ recommendation of the general practitioner
2. ☐ recommendation of another doctor
3. ☐ recommendation of the family practice nurse
4. ☐ vaccination in a family member/friend
5. ☐ media campaign (TV, radio, press)
6. ☐ vaccination was refunded (free of charge)
7. ☐ other (please, specify)………………………….

10. Have you ever experienced side effects after taking the flu vaccine?

1. ☐ yes
2. ☐ no – if „no" please, skip to question 12
3. ☐ I do not remember

11. What was the nature of the side effects?

1. ☐ pain at the injection site
2. ☐ redness at the injection site
3. ☐ swelling at the injection site
4. ☐ fever
5. ☐ musculoskeletal pain
6. ☐ general malaise
7. ☐ anaphylactic shock
8. ☐ other (please, specify) ……………………………

12. Are you going to get a flu shot next epidemic season?

1. ☐ yes
2. ☐ no
3. ☐ I don't know
4. ☐ only when vaccination is refunded (free of charge)

III. KNOWLEDGE ABOUT INFLUENZA

13. What sources of information about influenza do you use?

1. ☐ GP
2. ☐ another doctor
3. ☐ family practice nurse
4. ☐ family/friends
5. ☐ media campaigns (TV, radio, press, Internet)
6. ☐ other (please, specify) ………………………..

14. Influenza is a disease caused by:

1. ☐ bacteria
2. ☐ virus
3. ☐ it is a mixed infection
4. ☐ I do not know

15. How many people worldwide die annually from the flu?

1. ☐ approx. 10,000
2. ☐ approx. 100,000
3. ☐ approx. 0.5 million
4. ☐ I do not know

16. Are flu and common cold the same disease?

1. ☐ yes
2. ☐ no
3. ☐ I do not know

17. Some otherwise healthy adults may be able to infect others beginning one day before influenza symptoms develop

1. ☐ true
2. ☐ false
3. ☐ I do not know

18. Routes of influenza transmission are as follows:

1. ☐ coughing and sneezing
2. ☐ coughing, sneezing and by contact with contaminated objects
3. ☐ contact with blood
4. ☐ I do not know

19. Complications of influenza include:

1. ☐ pneumonia and bronchitis
2. ☐ otitis media
3. ☐ sinusitis
4. ☐ inflammation of the heart muscle and pericardium

5. ☐ encephalitis and meningitis
6. ☐ all of the above
7. ☐ I do not know

20. People at higher risk of severe influenza complications:

1. ☐ young children
2. ☐ pregnant women
3. ☐ people 65 years and older
4. ☐ all the above mentioned
5. ☐ I do not know

21. Can the flu become life-threatening?

1. ☐ yes
2. ☐ no
3. ☐ I do not know

22. Some people should not receive influenza vaccines at all:

1. ☐ true
2. ☐ false
3. ☐ I do not know

23. To effectively protect against the disease flu vaccine should be received:

1. ☐ once a year
2. ☐ more than once a year
3. ☐ one vaccination gives immunity for life
4. ☐ I do not know

24. To provide the optimal protection against infection a flu shot should be administered:

1. ☐ at the beginning of autumn
2. ☐ in winter
3. ☐ it doesn't matter
4. ☐ I do not know

25. Effective methods of protection against influenza include all except:

1. ☐ frequent hand washing
2. ☐ avoiding crowds during the influenza season
3. ☐ flu vaccination
4. ☐ home remedies, for example: raspberry juice, drink with honey and lemon
5. ☐ I do not know

26. Some animals, such as birds, horses, pigs, can be a source of influenza infection for humans

1. ☐ yes
2. ☐ no
3. ☐ I do not know

THANK YOU FOR COMPLETING THE QUESTIONNAIRE
